# The Use of Emerging Technologies in Alcohol Treatment

**Published:** 2011

**Authors:** John A. Cunningham, Kypros Kypri, Jim McCambridge

**Keywords:** Alcohol use, abuse, and dependence, problem drinking, treatment, intervention, technology, emerging technology, computer technology, Internet, telecommunication, text messaging

## Abstract

Emerging technologies, such as the Internet and text messaging, have an ever-growing role in providing services to problem drinkers. This article summarizes selected examples of emerging technologies that have been developed and implemented as stand-alone interventions and as part of other face-to-face interventions. It provides a taste of the different opportunities available for implementing emerging technologies as a way to improve the accessibility and effectiveness of services for problem drinkers.

How can the greatest number of problem drinkers—an intentionally broad term covering the range of drinking behaviors—obtain help given limited health care resources? Emerging technologies such as electronic tools that service providers can use to help problem drinkers may provide a partial answer to this question. This article will outline the rationale for using emerging technologies to help problem drinkers and summarize the types of technologies already being used, along with a review of the research base supporting their use. Some of the technologies mentioned in this article, such as the Internet, are fairly new, whereas others, such as telephones, have been in existence for some time, but all have only recently been applied to the treatment of alcohol problems. Several overviews of this topic already have been published (Bewick et al. 2008; Carey et al. 2009; Hester and Miller 2006; Kypri et al. 2005), and, because the state of technology is moving so rapidly, the intent of this article is not to provide a systematic review of all the research done in this area. Rather, the summary seeks to provide key examples and commentary on what has been done so far in order to stimulate the reader to consider ways that emerging technologies might be incorporated into one’s own practice.

Emerging technologies may have several advantages over traditional methods in promoting quality care for problem drinkers. First, they may increase access to evidence-based treatment to a larger number of people. Currently, most people with alcohol problems never seek face-to-face assistance, whether from a specialized treatment agency, from their general practitioner, or even from groups such as Alcoholics Anonymous. Indeed, it is estimated that in Canada and the United States only between 1 in 3 and 1 in 14 people with a drinking problem seek treatment (Burton and Williamson 1995; Cunningham and Breslin 2004; Hasin 1994; Roizen et al. 1978). Common reasons for not seeking treatment include stigma, embarrassment, and a desire to handle problems on their own (Cunningham et al. 1993; Grant 1997; Roizen 1977; Sobell et al. 1992; Tuchfeld 1976). In addition, many people have difficulty accessing treatment, even where there are comprehensive nationwide services, because they live in rural areas, far from any treatment services. Emerging technologies, particularly those delivered over the Internet, have the potential to overcome some of these barriers.

Emerging technologies also may be utilized by segments of the general population traditionally underserved in face-to-face treatment. Specifically, a larger proportion of female and older adults access Internet-based interventions (IBIs) than are typically seen in traditional treatment contexts (Cunningham et al. 2000; Humphreys and Klaw 2001). Taken together, the potential for emerging technologies to supplement standard treatment approaches in a cost efficient fashion, and to promote access to those traditionally underserved by our current treatment options, makes this an exciting area for research and development. Recognizing this potential, much research is currently underway.

## Definitions

Before moving to a review of emerging technologies and their current research base, it is worthwhile to briefly define some terms. First, the vague term “problem drinkers” was intentionally used to cover the range of drinkers from those who experience a few consequences and drink beyond recommended levels to those who could be defined as alcohol dependent. This term is used because many of the studies we review do not clearly define the severity of study participants’ drinking problems. It is safe to say that participants in research projects which involve interventions without face-to-face contact generally have less severe problems than patients clinicians typically see in addictions treatment settings (although some will certainly be as severe). An excellent quote by Heather (1989, p. 366) on brief interventions in community settings is as applicable today concerning emerging technologies as it was two decades ago about other types of brief interventions: “Thus the evidence shows that brief interventions are effective and should be used for those individuals who are not actively seeking help at specialist agencies. This justification is, again, independent of level of seriousness, although most recipients of community-based brief interventions will obviously have problems of a less severe variety. Moreover, when potential clients are not actively seeking help, then the cost-effectiveness kind of argument does become relevant and it is ethically legitimate to ask what is the least expensive way of reaching the greatest number of smokers or excessive drinkers, etc.”

It also is necessary to define what is meant by treatment in relation to emerging technologies. In this article, interventions are roughly classified by length, using the catch phrase “screeners” to describe brief interventions that are approximately 10 minutes long and “cognitive–behavioral treatments” to describe multisession interventions. It should be noted that these distinctions represent more of a continuum than two distinct categories, because some screeners are quite extensive and some cognitive–behavioral treatments can be quite brief if the participant so chooses. Also, all the interventions discussed in the article can be thought of as different types of treatment if treatment is defined as something that helps problem drinkers reduce the amount they drink. In fact, one of the exciting things about IBIs is that there is quite a lot of evidence such that even brief 10-minute screeners can reduce problem drinkers’ alcohol consumption. Among the different technologies, some are designed to be used primarily in isolation, without contact with face-to-face services, and others are typically used in more traditional face-to-face settings.

## Emerging Technologies for Use in Isolation

Although some programs can be delivered by a CD-ROM for use on a personal computer or preinstalled computer software programs (Hester and Delaney 1997; Schinke et al. 2004), the majority of these programs take advantage of the Internet for their delivery. (For a summary of these Internet-based tools, see the textbox.)

### Screeners

Online screeners represent the most common IBIs for problem drinkers. They typically ask participants a generally short series of questions about their drinking and then provide personalized feedback summaries that illustrate their risk of experiencing an alcohol-related problem. For example, www.AlcoholScreening.org (Saitz et al. 2004) is a very popular free-access intervention that uses the Alcohol Use Disorders Identification Test (AUDIT). The World Health Organization developed AUDIT as a brief, 10-item screener that provides an indication of the severity of a participant’s alcohol concerns (Babor et al. 1989). In the United States, the site AlcoholScreening.org links participants to an online list of treatment resources that allows them to find services in their community, matched by ZIP Code, if they are concerned by the results of their AUDIT test. This illustrates the potential for Internet technologies to integrate or combine both online and offline intervention components.

A number of other online screening tests are available for general population use. For example, www.DrinkersCheckup.com (Hester et al. 2005) provides a Web adaptation of the popular in-person Drinker’s Check-up (Miller et al. 1988), which research shows works well in a face-to-face setting. However, research has not yet evaluated whether this intervention works when it is delivered over the Internet, so caution should be taken in assuming that it will work in settings with no personal contact.

Research has evaluated the Check Your Drinking (CYD) screener, available at www.CheckYourDrinking.net (Cunningham et al. 2006). This intervention not only provides feedback on participants’ drinking but compares it with drinking by others of the same age, gender, and country of origin (for the United States, Canada, and the United Kingdom to date) (see the figure for an example of this kind of normative feedback). Three randomized controlled trials tested whether this intervention worked in a face-to-face setting (Doumas and Hannah 2008; Doumas and Haustveit 2008; Doumas et al. 2009), and another tested how well it worked when participants accessed the intervention via the Internet from their own homes or another location of their choice (Cunningham et al. 2010). The studies showed that participants reduced their drinking in both settings.

Although the screeners discussed above are available to the general public, much of the work on IBIs, and on personalized feedback screeners in particular, has been done with college-student samples (Bewick et al. 2008; Elliott et al. 2008; Hallett et al. 2009; Saitz et al. 2007; Walters et al. 2005). As such, some screeners, such as www.eChug.com, are designed specifically for college students. Several randomized controlled trials have shown that e-Chug works well to reduce student drinking (Walters et al. 2007). Although there is a fee to view the e-Chug intervention on its home Web site, readers can see a working example by searching for University-based copies.

Other college-based IBIs focus on curbing drinking at specific events. For example, Neighbors and colleagues (2009) developed a successful normative feedback intervention to reduce harmful alcohol consumption on a student’s 21st birthday. The intervention included personalized feedback, based on information collected a week before the person’s birthday, which highlighted how much the person was planning to drink on his or her birthday compared with how much people usually drank on their 21st birthday. This study demonstrates the extent to which IBIs can be tailored and personalized to target situations as well as to the particular demographic and drinking characteristics of participants.

### Cognitive–Behavioral Treatment Programs

Several IBIs contain a wide range of cognitive behavioral tools to help problem drinkers. All of the programs mentioned below contain many of the tools that are commonly used in face-to-face settings such as drinking diaries, goal setting exercises and relapse-prevention techniques. A Dutch-language site, www.minderdrinken.nl, for example, which has been subjected to a randomized controlled trial, showed that people who used it reduced their drinking 6 months later (Riper et al. 2008). The U.K. Web site Down Your Drink (www.downyourdrink.org.uk) is a similar Web site and there is some research indicating it is helpful (Linke et al. 2007). Hester and colleagues (2009) reported that a drinking-in-moderation training program provided as an adjunct to an online moderation management group appeared to have promising results. However, because this trial involved some face-to-face interaction, it is unclear what influence the program would have in an Internet-only setting.

Other Web sites have been developed based on cognitive–behavioral tools used in brief interventions, including the Alcohol Help Center (www.AlcoholHelpCenter.net). However, although the tools contained in this Web site have a solid research base, the Web site itself has not been subjected to a randomized controlled trial, so its efficacy in helping problem drinkers in an online context has not yet been demonstrated. Finally, there are some excellent trials that have demonstrated the efficacy of computer-based interventions that are not delivered over the Internet (Hester and Delaney 1997; Schinke et al. 2004).

### Other Emerging Technologies

A variety of other emerging technologies have potential to help problem drinkers outside of the traditional face-to-face settings. One is the telephone, which, although it is more than 100 years old, is relatively new as used in alcohol treatment interventions. For example, researchers have recently found that telephone-based counseling (Bischof et al. 2008; Mello et al. 2008) can be quite useful. Of course, mobile telephones, which allow text messaging and Internet access, are an altogether more modern phenomenon, but there is surprisingly little research on interventions primarily delivered in the form of phone-based discussions or automated responses. There is, however, some evidence that text messaging can be used to encourage smoking cessation (Rodgers et al. 2005).

There also has been success with alcohol and drug counseling done online in real time, rather than face-to-face, allowing access to people who might not otherwise have access (Swan and Tyssen 2009). Other online cognitive–behavioral programs incorporate text message and e-mail components to promote continued contact with participants (e.g., see “Tools” section of www.AlcoholHelpCenter.net). However, the use of these tools still is in its infancy and has little or no research base demonstrating its efficacy with problem drinkers (although there have been trials conducted in the area of tobacco control with promising results [Brendryen and Kraft 2008]).

**Examples of Internet-Based Interventions**

These Web sites are examples of different Internet-based interventions that can be viewed free of charge.

**General Population Screeners**

- **http://rethinkingdrinking.niaaa.nih.gov** (Produced by the National Institute on Alcohol Abuse and Alcoholism.)
- **www.CheckYourDrinking.net** (Produced by a for-profit eHealth company. Personalized versions are available for a fee.)
- **www.AlcoholScreening.org** (Produced by a nonprofit organization.)
- **www.DrinkersCheckup.com** (This intervention is somewhere in length between a screener and a cognitive-behavioral intervention.)

**College Student Screeners**

- **www.CheckYourDrinkingU.net** (Produced by a for-profit eHealth company. Personalized versions of this Web site are available for a fee.)
- **www.eChug.com** (Produced by a for-profit eHealth company. This version is not available free-of-charge, but interested readers can readily find a free-access version through an Internet search. Personalized versions of this Web site are available for a fee.)

**Support Groups**

- **http://aa-intergroup.org** (Produced by Alcoholics Anonymous)
- **http://www.moderation.org** (Produced by Moderation Management)
- **www.AlcoholHelpCenter.net** (Produced by a for-profit eHealth company. This version contains an online support group. Personalized versions are available for a fee.)

**Cognitive-Behavioral Interventions**

- **www.AlcoholHelpCenter.net** (Produced by a for-profit eHealth company. Personalized versions are available for a fee.)
- **www.DownYourDrink.org.uk** (Produced by a nonprofit organization. This Web site is situated in the United Kingdom, so some drinking terminology (e.g., drink sizes) is different from North America.)

Online support groups also can be used to help problem drinkers. Many common support groups, including Alcoholics Anonymous and Moderation Management, have an online counterpart (http://aa-intergroup.org and www.moderation.org, respectively). Research on the utilization of online support groups on their own (Cunningham et al. 2008; Humphreys and Klaw 2001) and in conjunction with other IBIs (Hester et al. 2009) has found that, although not everyone will choose to use online groups, they can be a good source of support for those who do take advantage of them.

Some IBIs target specific population groups, including women living in rural areas (Finfgeld-Connett et al. (2008) and members of the military (Williams et al. 2009). Studies of these targeted interventions show promise, and it is likely that IBIs specialized for particular target groups will be an emerging area over the next several years (e.g., Schinke et al. 2009).

## Emerging Technologies for Use in Face-to-Face Settings

In large part, the types of emerging technologies for use in face-to-face settings mirror those found for the no contact interventions summarized above.

### Screeners and Cognitive–Behavioral Treatment Programs

Treatment providers are beginning to recognize that computerized, and largely Internet-based, interventions can be useful even in settings where patients see providers face to face. Kypri and colleagues (2008) found that it is useful and efficient to use a computerized screener to provide patients seeking treatment from their primary care providers with feedback about their drinking (also see, Linke et al. 2005; Meyer et al. 2008). Others have used a normative feedback screener to assess the drinking norms of a live group, such as an athletics team, and then provide feedback based on the combined responses of the group (LaBrie et al. 2008, 2009).

## Emerging Technologies for Use in Different Settings

Some research has examined using computerized interventions in specialized settings, including the emergency department in hospitals (Maio et al. 2005), prenatal clinics (Armstrong et al. 2009), and schools (Newton et al. 2009), as well as to help provide services to people with concurrent alcohol problems and mental health concerns (Kay-Lambkin et al. 2009). In addition, research suggests that emerging technologies streamline and improve work done in addiction treatment settings (Carroll et al. 2008; Vahabzadeh et al. 2009) and the automated collection of follow-up data (Helzer et al. 2008).

## Research Methods

The strength of any conclusions that can be made about the efficacy of different interventions will largely depend on the research designs employed to evaluate emerging technologies used to treat problem drinking (Cunningham and van Mierlo 2009; Murray et al. 2009). As with many research questions, the best way to study whether an intervention works is by conducting a randomized controlled trial. Without that type of design, it is difficult to draw conclusions about the cause of any effect seen.

Some study design elements are of particular relevance to studies investigating emerging technologies. For example, in IBI research attrition bias can be highly problematic. It is not unusual for IBI trials to retain only 35 to 40 percent of participants at follow-up. This is so common that it has even been labeled “the law of attrition” (Eysenbach 2005). These levels of loss to follow-up seriously jeopardize the reliability of treatment effect estimates, particularly if different treatment groups have different attrition rates. There are evidence-based measures, such as providing incentives for participants who respond to research follow-ups, that, though expensive, can improve participant retention rates. As such, researchers should consider using them to increase the value of their studies.

Finally, studies can be more or less informative depending on whether they examine the efficacy of an intervention—often undertaken in more artificial research conditions—or the effectiveness of an intervention—undertaken in more real-world–intended application conditions. In IBI research, for example, if an intervention is designed to be administered over the Internet, a research trial in which the intervention is delivered in a face-to-face setting has limited utility. This is because people may comply with an intervention when they are confined within the setting of a face-to-face laboratory but may easily get bored and move on to some other Web site when they look at the same intervention online in the privacy of their own homes (Cunningham and van Mierlo 2009; Danaher et al. 2005). Thus, it is important to consider whether the emerging technology under consideration for adoption was actually evaluated in a setting similar to the one in which it will be used.

## Future Directions

In looking at emerging technologies and the treatment of problem drinking, it is clear that there is a wide range of modalities and settings in which these technologies can be applied. This article only has examined a small subset of research-supported applications that include Internet- and computer program–based screeners and full intervention programs as well as telephone-, e-mail–, and text message– based applications. Similarly, it has highlighted interventions that are delivered over the Internet via online social support groups, in primary care settings, in emergency departments, in prenatal care services, and in schools. The key theme is that most, if not all, emerging technologies that provide new ways for people to communicate with each other can be used to deliver help to problem drinkers. In addition, each of these technologies has the potential to be used in specialized treatment, general health care, or other social service settings to promote accessibility, effectiveness, and cost efficiency of treatment.

Also apparent from this overview is the extent to which these emerging technologies still are in their infancy. Much work needs to be done in order to demonstrate that these tools work both in theory and in real-world settings and to explore how they can be used most effectively. It also will be important to develop a way to distinguish, for the benefit of consumers, evidence-based IBIs from untested IBIs and to study ways to engage problem drinkers with effective technology-based interventions.

One final challenge will be to explore how to integrate these new treatment modalities into traditional face-to-face treatment—identifying where this is worth doing and where it is not. There is longstanding recognition that it is important to develop a continuum of treatment opportunities for problem drinkers (Institute of Medicine 1990). Emerging technologies are proving to be one tile in this larger mosaic of treatment services for those with alcohol concerns.

## Figures and Tables

**Figure:**
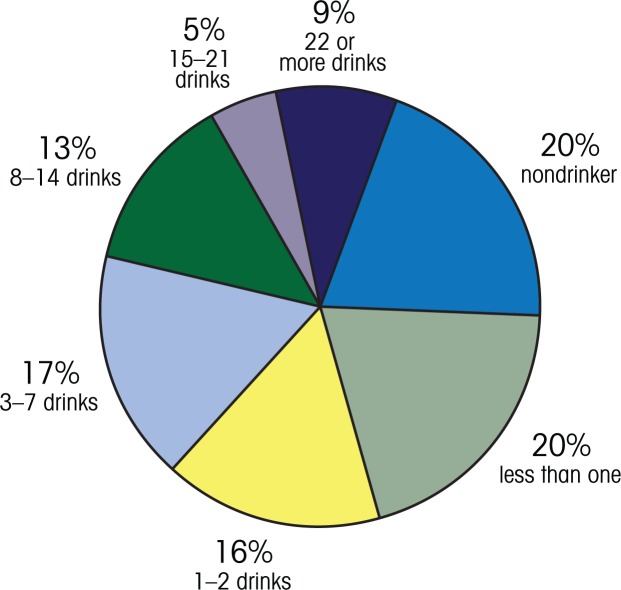
Average drinks per week for males aged 21–24 from the United States. Example feedback from the Check Your Drinking screener: www.CheckYourDrinking.net. How do you compare to males your age from the United States? The highlighted slice of the pie chart below is where your drinking fits compared with other males in your age range from United States. SOURCE: www.checkyourdrinking.net
